# Bloodless Method of Amputation, Case of

**Published:** 1874-04

**Authors:** 


Trial of the Bloodless Method of Operating in Mercy Hospital, Chicago.—Esmarch, of Germany, has recently proposed a method of saving blood in operations upon the extremities, which is attracting great attention in Europe. As it is a novelty, the following trial of it at the Mercy Hospital clinic, by Professor Andrews, will be read with interest:

The patient was a large-framed young woman, suffering with incurable neuralgic cicatrices in the left foot, following the cure of extensive caries of the tarsal and metatarsal bones. Every other resource of medicine and surgery having been exhausted, it was determined to remove the offending part by Pirogoff's amputation.

Anæsthetics being administered, Professor Andrews took a six-yard roller of elastic bandage, and, commencing at the toes, applied it tightly, lapping each turn over half the previous one, until it extended four inches above the knee. A piece of common rubber tubing, about half an inch in exterior diameter, and four yards long, was then wound several times very tightly around the thigh, just at the upper edge of the elastic bandage, and secured by tying its ends together. The effect of these manipulations was, that the elastic bandage expelled all the blood from the limb by its great pressure, while the girdle of rubber tubing acted as a tourniquet to prevent its return. The elastic bandage was now removed, when the limb appeared blanched and somewhat shrunken. The amputation was performed rather slowly and deliberately, so as to test the efficiency of the new plan. The incisions yielded not a stain of blood, except at one point, where three or four drops were visible; at every other place they were as bloodless as they would have been on a stick of wood, so that the minutest dissections could have been made as easily as upon the dead subject. It was only after the incisions were over, and the tubing was loosened in order to ascertain the position of arterial twigs requiring ligatures, that any oozing of blood occurred.

